# Acupuncture in Autism Spectrum Disorder: A Narrative Review of Neurotransmitter Regulation and Neuroplasticity

**DOI:** 10.3390/biomedicines14081701

**Published:** 2026-07-29

**Authors:** Anjali Kariyarath Valappil, Seung-Nam Kim

**Affiliations:** Department of Meridian and Acupoint, College of Korean Medicine, Dongguk University, Goyang 10326, Republic of Korea

**Keywords:** autism spectrum disorder, acupuncture, neuroplasticity, GABA, BDNF, monoamines

## Abstract

Autism spectrum disorder (ASD) is a heterogeneous neurodevelopmental condition characterized by impairments in social communication, restricted and repetitive behaviors, sensory dysregulation, and frequent psychiatric comorbidities. Increasing attention has been directed toward acupuncture as a complementary neuro-modulatory intervention; however, its underlying molecular mechanisms remain incompletely understood. This review synthesizes evidence from preclinical, clinical, and molecular studies published between 2015 and 2025 to examine how acupuncture influences neurobiological pathways relevant to ASD. Current evidence indicates that acupuncture modulates multiple neurotransmitter systems, including glutamatergic, GABAergic, dopaminergic, serotonergic, and noradrenergic signaling, while also influencing neurotrophin-mediated plasticity, neuroinflammatory responses, and synaptic function. Studies conducted directly in ASD models demonstrate regulation of excitatory/inhibitory balance, monoaminergic signaling, neurotrophin pathways, and ASD-associated behavioral outcomes, whereas evidence from related neuropsychiatric conditions provides complementary mechanistic support for these pathways. Collectively, the findings suggest that acupuncture may act through coordinated modulation of interconnected neurotransmitter and neuroplasticity networks rather than a single molecular target. However, direct mechanistic evidence in ASD-specific models and clinical populations remains limited, and considerable heterogeneity exists among acupuncture protocols and outcome measures. Future studies integrating standardized stimulation paradigms with molecular, electrophysiological, neuroimaging, and behavioral assessments will be essential to validate the proposed mechanisms and clarify the translational potential of acupuncture in ASD.

## 1. Introduction

Autism spectrum disorder (ASD) is a complex neurodevelopmental condition characterized by persistent impairments in social communication and interaction, together with restricted and repetitive patterns of behavior and atypical sensory processing [1,2]. The global prevalence of ASD is estimated at approximately 1–2% of children in many countries, and despite advances in early intervention, many individuals continue to experience substantial functional limitations and lifelong disability [3]. This enduring burden underscores an urgent need for innovative therapeutic strategies that go beyond conventional behavioral and pharmacological approaches.

Beyond the core features of ASD, a high proportion of individuals with ASD experience additional medical, psychological or developmental conditions (comorbidities). These frequently include intellectual disability, attention deficit/hyperactivity disorder (ADHD), anxiety disorders, mood disorders, epilepsy/seizures and sleep disturbances [4]. Importantly, many of these comorbidities share disrupted mechanisms, such as dysregulated neurotransmitter systems, impaired synaptic plasticity and altered neural circuit connectivity, which overlap with those implicated in ASD core pathology [5,6,7,8,9]. This convergence suggests that interventions capable of modulating neurotransmitters and neuroplasticity may have broad relevance within the ASD phenotype and its comorbid conditions.

ASD is currently managed using behavioral interventions, educational support, pharmacotherapy for associated symptoms, and, in selected cases, complementary therapies. However, no pharmacological treatment directly targets the core social communication deficits of ASD, and currently approved medications primarily address associated symptoms such as irritability, hyperactivity, or anxiety, often accompanied by adverse effects [10]. Consequently, there is growing interest in complementary therapeutic approaches that may modulate underlying neurobiological mechanisms with fewer systemic side effects [11]. Among these, acupuncture has emerged as a promising neuro-modulatory intervention owing to its reported effects on neurotransmitter regulation, neuroplasticity, and neuroimmune signaling. Nevertheless, the molecular mechanisms by which acupuncture may influence ASD-related neurobiology remain incompletely understood.

Rooted in traditional East Asian medicine, acupuncture has been further shown in multiple experimental and clinical studies to shift excitatory/inhibitory balance in the brain [12,13]. Though few studies to date have examined acupuncture specifically in ASD, the known mechanisms of acupuncture correspond strongly to the neurobiological pathways central to ASD and its comorbidities, specifically, neurotransmitter modulation (e.g., glutamate, GABA, dopamine, serotonin, oxytocin) [14,15], neurotrophic factor activation (e.g., BDNF/TrkB) and downstream signaling via PI3K/Akt, MEK/ERK and mTOR. These intersections provide a compelling rationale to explore acupuncture as an adjunctive intervention in ASD.

A previous Cochrane review by Ref. [16] primarily evaluated the clinical efficacy and safety of acupuncture for ASD based on the limited clinical evidence available at that time. In contrast, the present review focuses on advances in the neurobiological mechanisms underlying acupuncture reported primarily between 2015 and 2025, integrating evidence from preclinical models, molecular studies, neurotransmitter regulation, neurotrophin signaling, synaptic plasticity, and neuroimmune pathways to provide an updated mechanistic framework for understanding acupuncture in ASD.

This review focuses on the mechanistic actions of acupuncture on core neurobiological features of ASD and its common comorbidities, particularly neurotransmitter regulation, synaptic plasticity, and related intracellular signaling pathways. Drawing on converging evidence from recent animal models and clinical studies, it outlines a mechanistic framework that can guide future research.

## 2. Literature Search Strategy

A literature search was conducted using PubMed, Scopus, and Web of Science to identify studies investigating the neurobiological effects of acupuncture in ASD and related conditions. Searches primarily included studies published between January 2015 and December 2025 using combinations of the keywords “autism spectrum disorder”, “ASD”, “acupuncture”, “electroacupuncture”, “laser acupuncture”, “autism”, “norepinephrine”, “neurotransmitter”, “glutamate”, “GABA”, “dopamine”, “serotonin”, “BDNF”, and “neuroplasticity”. Original research articles, preclinical animal studies, clinical studies, and relevant review articles published in English were considered. Given the limited availability of mechanistic acupuncture studies conducted directly in ASD models, studies involving related neuropsychiatric or neurodevelopmental conditions, including depression, anxiety, epilepsy, and insomnia, were also included when they investigated molecular or neurobiological pathways relevant to ASD. Studies were excluded if they lacked measurable neurobiological, molecular, or electrophysiological outcomes or had no mechanistic relevance to ASD.

Although the literature search primarily focused on studies published between 2015 and 2025, a small number of earlier publications were retained where they provided foundational mechanistic evidence not superseded by the more recent literature, including Connors et al. on plasma serotonin abnormalities in ASD [17], and Park et al. and Zhou et al. on acupuncture-related serotonergic [18] and GABAergic mechanisms [19], which remain frequently cited reference points in this field.

## 3. Regulation of Neurotransmitters

### 3.1. Excitatory/Inhibitory Balance (Glutamate/GABA)

The balance between excitatory glutamatergic (GLU) signaling and inhibitory γ-aminobutyric acid (GABA) signaling is essential for maintaining synaptic homeostasis, regulating neural oscillations, and shaping experience-dependent plasticity in the developing and mature brain. Within this system, glutamate acts primarily through ionotropic (NMDA, AMPA) and metabotropic (mGluR1-8) receptors to induce depolarization and promote synaptic strengthening, whereas GABA, synthesized by glutamate decarboxylase isoforms GAD65 and GAD67, activates GABA_A_ (ionotropic) and GABA_B_ (metabotropic) receptors to mediate hyperpolarization and inhibitory control of neuronal firing. The coordinated regulation of these pathways depends on receptor subunit composition (e.g., GABR_A_1, GABR_B_3, GRIN2B), synaptic scaffold proteins (e.g., SHANK, gephyrin), interneuron integrity (particularly parvalbumin-positive fast-spiking interneurons), and transporters that maintain extracellular neurotransmitter clearance (EAATs for glutamate, GATs for GABA).

Mounting evidence indicates that this system is disrupted in ASD, with convergent findings from transcriptomics, magnetic resonance spectroscopy (MRS), and post-mortem neuropathology demonstrating reduced GABAergic tone and/or excessive glutamatergic activity across multiple cortical and subcortical regions [20]. Post-mortem studies consistently report reductions in GAD65/GAD67 levels, decreased expression of GABA_A_ receptor subunits, especially GABR_B_3, a high-confidence ASD gene and loss or functional impairment of parvalbumin (PV) interneurons, which are central to inhibitory control and gamma-band synchrony. Correspondingly, MRS studies show lowered GABA concentrations and elevated Glu/GABA ratios in the auditory cortex, motor cortex, and anterior cingulate cortex of children and adults with ASD, indicating a shift toward cortical hyperexcitability. Genetically, ASD-associated variants in SHANK3, NRXN1, CNTNAP2, GRIN2B, and SLC6A1 further implicate synaptic scaffolding, receptor signaling, and neurotransmitter transport in E/I imbalance.

Dysregulation of E/I balance is closely linked to core ASD phenotypes, including social communication differences and sensory hypersensitivity, and similar network-level alterations are reported in conditions that frequently co-occur with ASD, such as epilepsy, anxiety disorders, intellectual disability, and sleep disturbance. The high co-occurrence of ASD and epilepsy (up to 30%) is consistent with shared vulnerability at the level of inhibitory circuit regulation, although causal relationships remain unresolved. Developmental disruption of the GABAergic “excitatory-to-inhibitory” switch, mediated by NKCC1/KCC2 chloride transporter maturation, has been proposed as one mechanism contributing to altered critical-period plasticity and long-term circuit organization in ASD.

Direct evidence comes mainly from VPA-induced rat models of autism, where several groups report that acupuncture shifts GABA and glutamate signaling alongside behavioral improvement. For example, laser acupuncture at HT7 in a valproic acid (VPA)-induced rat model of autism demonstrated improved GABAergic function: specifically, treatment increased the expression of both GAD65 and GAD67 proteins together with Purkinje cell density and reduced GABA-transaminase (GABA-T) activity in the cerebellum, while also decreasing IL-6 and oxidative stress markers. These findings indicate that acupuncture has been reported to modulate GABAergic enzyme expression, cerebellar cellular integrity, and inflammatory markers in this model [14]. NRG1-ErbB4 signaling modulates the excitability of pyramidal neurons across key corticolimbic regions, including the medial prefrontal cortex (mPFC), hippocampus, and amygdala, by facilitating GABA release and maintaining E/I balance. Alterations in this pathway have been associated with ASD-related synaptic and behavioral phenotypes in experimental models. In a recent study by Huang et al., scalp acupuncture (GV24 and GB13) significantly ameliorated autism-like behaviors in VPA-induced ASD rat models, with concurrent normalization of the NRG1-ErbB4-GABAergic signaling axis [21]. Specifically, acupuncture upregulated NRG1 and ErbB4 expression in the mPFC and hippocampus, leading to improved social and exploratory behaviors These findings suggest potential modulation of the NRG1–ErbB4–GABAergic axis; however, the extent to which these molecular effects mediate behavioral outcomes remains unclear [21]. In a study by Chen et al., scalp acupuncture was applied to VPA rat models for four weeks and the RNA sequencing of the PFC revealed that acupuncture modulated hundreds of genes related to synaptic function, neural signaling, and immune responses [22].

**Figure 1:**
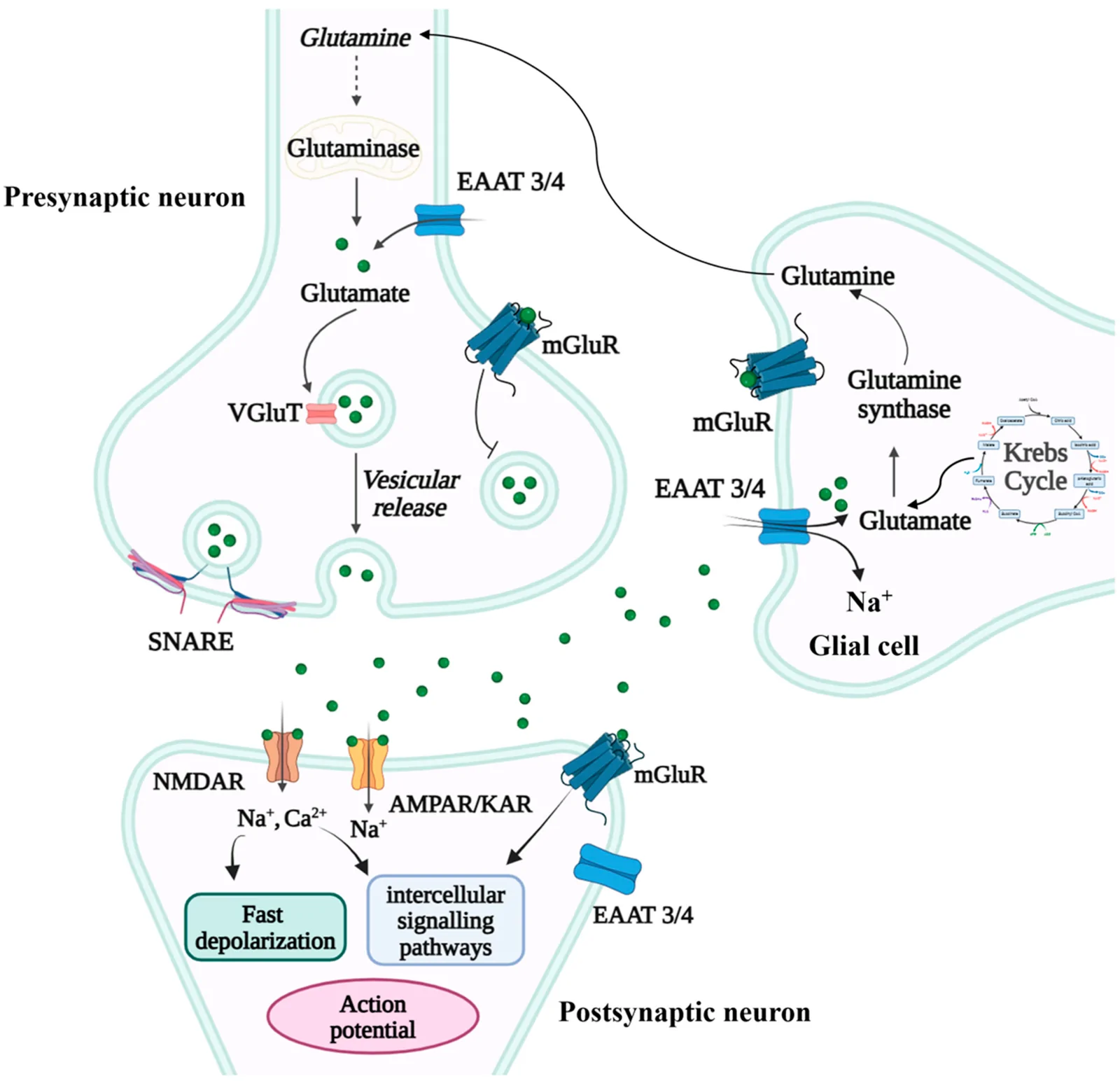
Glutamatergic neurotransmission and the glutamate–glutamine cycle. Schematic illustration of glutamatergic neurotransmission and the glutamate–glutamine cycle. In presynaptic glutamatergic neurons, glutamine is converted to glutamate (Glu) by glutaminase, after which glutamate is packaged into synaptic vesicles by the vesicular glutamate transporter (VGluT). Upon neuronal depolarization, vesicles fuse with the presynaptic membrane through the soluble N-ethylmaleimide-sensitive factor attachment protein receptor (SNARE) complex, releasing glutamate into the synaptic cleft. Released glutamate activates postsynaptic N-methyl-D-aspartate receptors (NMDARs), α-amino-3-hydroxy-5-methyl-4-isoxazolepropionic acid/kainate receptors (AMPAR/KAR), and metabotropic glutamate receptors (mGluRs), initiating membrane depolarization and intracellular signaling pathways that regulate neuronal excitability and synaptic plasticity. Glutamate is cleared primarily by excitatory amino acid transporters 3 and 4 (EAAT3/4) expressed on neurons and glial cells. Within glial cells, glutamate is converted back to glutamine by glutamine synthetase, whereas glutamate can also be replenished through intermediates of the Krebs cycle. Newly synthesized glutamine is transported back to presynaptic neurons to support continuous glutamate synthesis and maintain glutamatergic neurotransmission. Figure created with BioRender.com.

**Figure 2:**
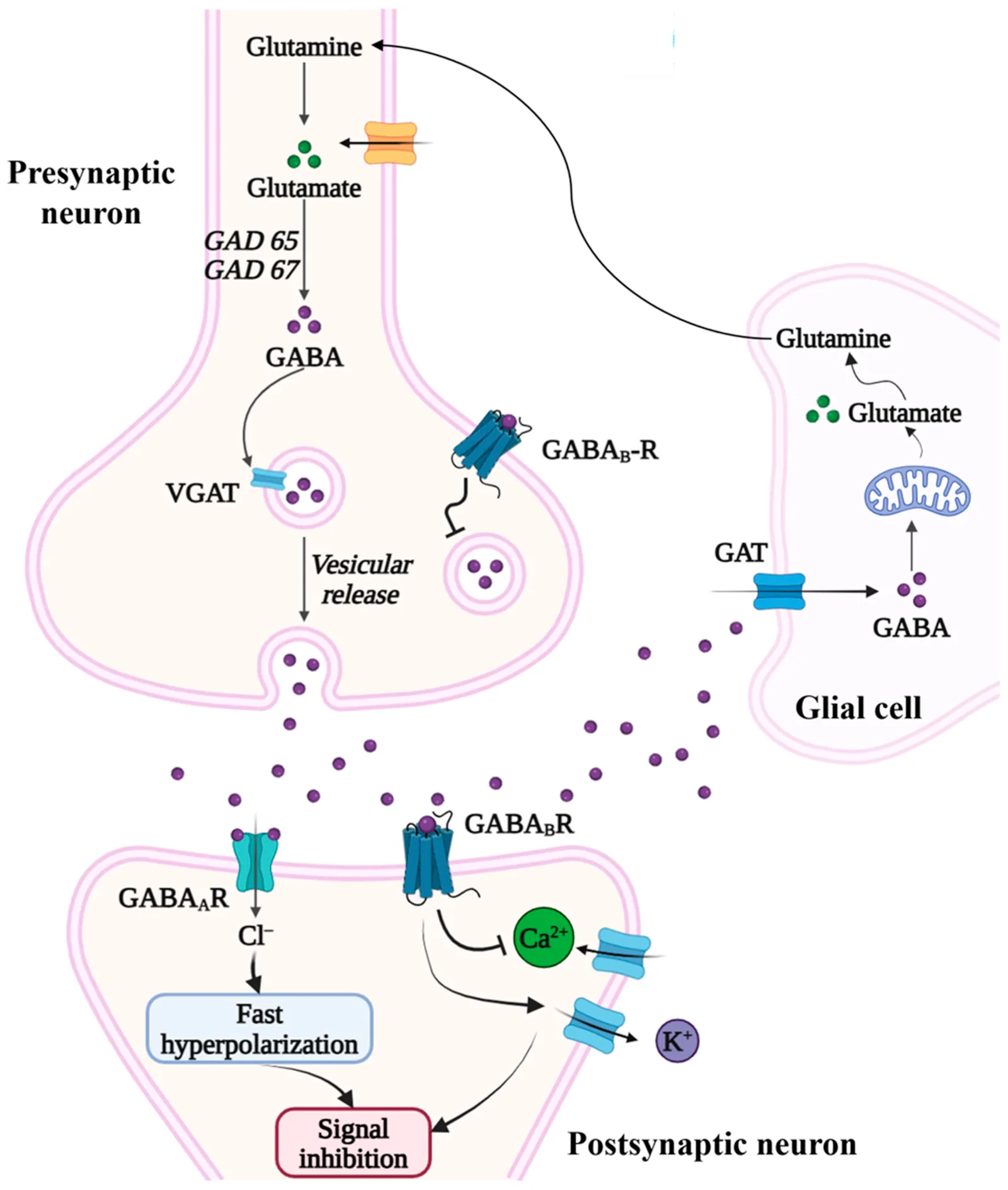
GABAergic neurotransmission and the glutamate–GABA cycle. Schematic illustration of γ-aminobutyric acid (GABA) neurotransmission and the glutamate–GABA cycle. In presynaptic GABAergic neurons, glutamate is converted to GABA by glutamate decarboxylase 65 and 67 (GAD65/GAD67). GABA is subsequently packaged into synaptic vesicles by the vesicular GABA transporter (VGAT) and released into the synaptic cleft following vesicular exocytosis. Synaptically released GABA binds to postsynaptic GABAA receptors (GABAA-R), which function as ligand-gated chloride (Cl^−^) channels to produce rapid membrane hyperpolarization, and GABAB receptors (GABAB-R), which are G protein-coupled receptors that suppress neuronal excitability by inhibiting calcium (Ca^2+^) influx and promoting potassium (K^+^) efflux. Presynaptic GABAB receptors also provide feedback inhibition of GABA release. Excess extracellular GABA is removed by the GABA transporter (GAT) and GABA transporter (GAT) expressed on neurons and glial cells. Within glial cells, GABA is metabolized and recycled through the glutamate–glutamine cycle, generating glutamate and glutamine that are transported back to presynaptic neurons to support continued synthesis of glutamate and GABA. This recycling pathway maintains inhibitory neurotransmission and contributes to the balance between excitatory and inhibitory signaling in the central nervous system. Figure created with BioRender.com.

Because direct ASD evidence of this kind is limited, additional support is drawn from preclinical work in epilepsy, insomnia, and stress-related model conditions that frequently co-occur with ASD and share elements of E/I circuit disruption. In a kainic acid rat model, EA reduced seizure frequency while upregulating GAD67 expression and increasing expression of glutamate transporters (EAATs), suggesting simultaneous enhancement of inhibition and glutamate clearance [23]. Similarly, in insomnia models, EA at HT7 and SP6 increased cortical GABA content and decreased Glu, accompanied by elevated expression of GABA_A_ receptor α1 subunit and downregulation of NMDA receptor NR2B, contributing to sleep regulation and reduced neuronal excitability [19]. Li et al. reported that electroacupuncture (EA) prevented depression-like behaviors in chronic unpredictable mild stress (CUMS) rats by enhancing GABA_B_ receptor activity and suppressing the NF-κB/NLRP3 inflammatory pathway, leading to reduced neuroinflammation and with potential implications for plasticity-related signaling pathways [24]. Across epilepsy, insomnia, and stress-related paradigms, acupuncture has been reported to influence components of synaptic transmission and inflammatory regulation. While these pathways overlap with mechanisms implicated in ASD, current evidence derives largely from non-ASD models (Figure 1 and Figure 2 and Figure S1).

Although alterations in synaptic scaffold proteins such as SHANK and gephyrin are strongly implicated in ASD, current evidence has not yet demonstrated whether acupuncture directly modulates these proteins. Future studies addressing this question would strengthen the mechanistic link between acupuncture-induced neurotransmitter regulation and synaptic remodeling.

### 3.2. Monoaminergic Systems

The monoaminergic system refers to a group of neurotransmitter systems in the brain that use monoamines, a class of neurotransmitters derived from aromatic amino acids. These systems are crucial for regulating mood, arousal, attention, motivation, and stress responses, and their dysfunction is linked to several neuropsychiatric disorders, including ASD, depression, ADHD, and anxiety. This system collectively maintains emotional balance and cognitive function through serotonin (5-HT), dopamine (DA), and norepinephrine signaling.

5-HT originates primarily from neurons in the dorsal and median raphe nuclei, which project broadly to the cortex, hippocampus, basal ganglia, cerebellum and spinal cord. It is synthesized from tryptophan via tryptophan hydroxylase and stored in vesicles, released into the synaptic cleft, and taken up by the serotonin transporter (SERT/5-HTT). Multiple receptor families (5-HT_1_ to 5-HT_7_) mediate its effects: for example, 5-HT_1_A modulates neuronal excitability and responds to stress, 5-HT_2_ receptors influence synaptic plasticity, and 5-HT_7_ regulates circadian rhythm and cognitive processes. 5-HT plays key roles in neurodevelopment (neurite outgrowth, synaptogenesis), sensory modulation, mood and anxiety regulation, sleep/wake cycles, aggression, and social behavior [17,25].

In ASD, there is substantial evidence of serotonergic dysfunction, including elevated whole-blood/platelet 5-HT in subgroups, altered serotonin transporter (SERT) availability, changes in receptor expression, and altered 5-HT neuronal development, all of which may contribute to social deficits, sensory hypersensitivity and repetitive behaviors [25,26,27,28]. Functionally, these serotonergic deficits correlate with social impairments, sensory atypicalities and repetitive behaviors in ASD [25]. Although specific human acupuncture studies in ASD measuring 5-HT parameters remain scarce, a recent animal study provides mechanistic insight: in a VPA-induced rat model of ASD, scalp acupuncture significantly improved social interaction, exploratory behavior and spatial memory. RNA sequencing of the hippocampus revealed that key serotonergic-related genes (HTR1_A_, HTR2_C_) were upregulated after acupuncture and hippocampal 5-HT concentration was restored to near control levels [15]. These findings suggest that acupuncture has been reported to modulate serotonergic signaling in these models. In models of depression or anxiety, acupuncture likewise has been shown to increase hippocampal 5-HT levels, enhance 5-HT1_A_ receptor expression, and reduce anxiety-like behavior, supporting translational relevance of this mechanism for ASD-related emotional and sleep disruptions [29,30]. Collectively, available data indicate that acupuncture may influence serotonergic tone and receptor-related signaling in preclinical settings; however, evidence for restored serotonergic homeostasis in ASD populations remains limited.

The DA system, encompassing mesocorticolimbic and nigrostriatal pathways, regulates reward processing, motivation, motor control, and executive function [31]. DA neurons in the ventral tegmental area (VTA) and substantia nigra pars compacta (SNc) project to the nucleus accumbens, amygdala, and prefrontal cortex, where dopamine acts via D1-like and D2-like receptors (DRD1-DRD5) to modulate synaptic excitability and reinforcement learning [32,33,34]. Dysregulation of DA circuits is strongly implicated in ASD and related disorders. Functional MRI and PET studies reveal altered striatal D2 receptor binding, lower dopamine D2/3 receptor (D2/3R) availability, and abnormal dopamine transporter (DAT, SLC6A3) density, particularly in individuals exhibiting stereotypic or reward-seeking behaviors [35,36,37,38]. Rodent ASD models (e.g., VPA, BTBR mice) show reduced mesocortical DA signaling and diminished social motivation, paralleling findings in ADHD and anhedonic depression [39].

Preclinical studies report that acupuncture has been associated with modulation of dopaminergic turnover, synthesis enzymes, and receptor-related gene expression in these circuits. In VPA-induced ASD rats, EA significantly enhanced DA levels and increased the expression of key biosynthetic enzymes, including tyrosine hydroxylase and aromatic L-amino acid decarboxylase in the PFC and hippocampus, which corresponded with improved learning, memory, and behavioral flexibility [40]. Similarly, a recent study using a Chinese acupuncture protocol demonstrated that repeated stimulation improved synaptic signaling in ASD rats, with RNA sequencing evidence revealing upregulation of dopaminergic synapse pathway genes, including Drd1, Drd2, Th, and SLC6A3 [22]. In maternally separated rat pups, acupuncture normalized DA turnover in the prefrontal–limbic circuitry, reversing stress-induced elevations in DA metabolites and restoring cortical–amygdala dopaminergic balance [41]. In a clinical trial in older adults with insomnia, EA at Baihui (GV20) and Yintang (EX-HN3) significantly increased serum DA levels compared with sham EA, demonstrating that acupuncture can upregulate systemic dopaminergic tone in humans as well [42].

Collectively, these findings indicate that acupuncture has been reported to modulate dopaminergic parameters at molecular and regional levels in experimental models. Whether these effects translate into durable circuit-level reorganization in ASD remains uncertain and warrants further investigation.

The noradrenergic system plays a pivotal role in regulating arousal, attention, and behavioral responsiveness. Its neurons project broadly across the central nervous system, allowing for this network to influence diverse functions ranging from sensory processing to cognition. Through this widespread innervation, the system regulates stress, helps sustain wakefulness and promotes a state of alert readiness, enabling efficient detection and evaluation of significant environmental cues [43,44]. A key hub of this network is the locus coeruleus (LC), the major source of norepinephrine (NE) in the brain. Activity within the LC-NE pathway has long been recognized as a fundamental driver of attentional control, acting almost like an internal regulator that tunes the brain’s capacity to focus, shift, and prioritize information [45]. NE acts via α- and β-adrenergic receptors to regulate cortical excitability, modulate synaptic plasticity, and integrate autonomic and emotional responses [46].

Recent perspectives further suggest that disruptions in LC–NE signaling may contribute to neurodevelopmental conditions. For instance, altered activity patterns within this system have been proposed to weaken attention-related processes in autism spectrum disorder, potentially influencing social orienting and engagement [47]. Unlike the other mechanistic pathways discussed above, current evidence for acupuncture-mediated modulation of noradrenergic signaling is derived exclusively from non-ASD models, including stress, insomnia, and anxiety paradigms. Therefore, its relevance to ASD remains inferential and requires direct validation in ASD-specific models and clinical studies. Recent experimental studies report modulation of noradrenergic signaling following acupuncture in stress-related paradigms through both direct neurotransmitter regulation and upstream neuroplasticity-associated mechanisms. In an acute restraint stress model, manual acupuncture (MA) at PC6 and HT7 significantly reduced elevated NE and its metabolite MHPG within the central amygdala, indicating suppression of stress-induced noradrenergic hyperactivity in limbic circuits governing anxiety and emotional reactivity [48]. Complementary findings were reported in a chronic stress-induced depression model where electroacupuncture restored monoaminergic balance by increasing hippocampal 5-HT while normalizing reduced NE levels; these changes occurred alongside suppression of cGAS-STING-NLRP3-mediated neuroinflammation, suggesting that acupuncture stabilizes monoaminergic transmission through anti-inflammatory and neuroprotective pathways [49].

**Figure 3:**
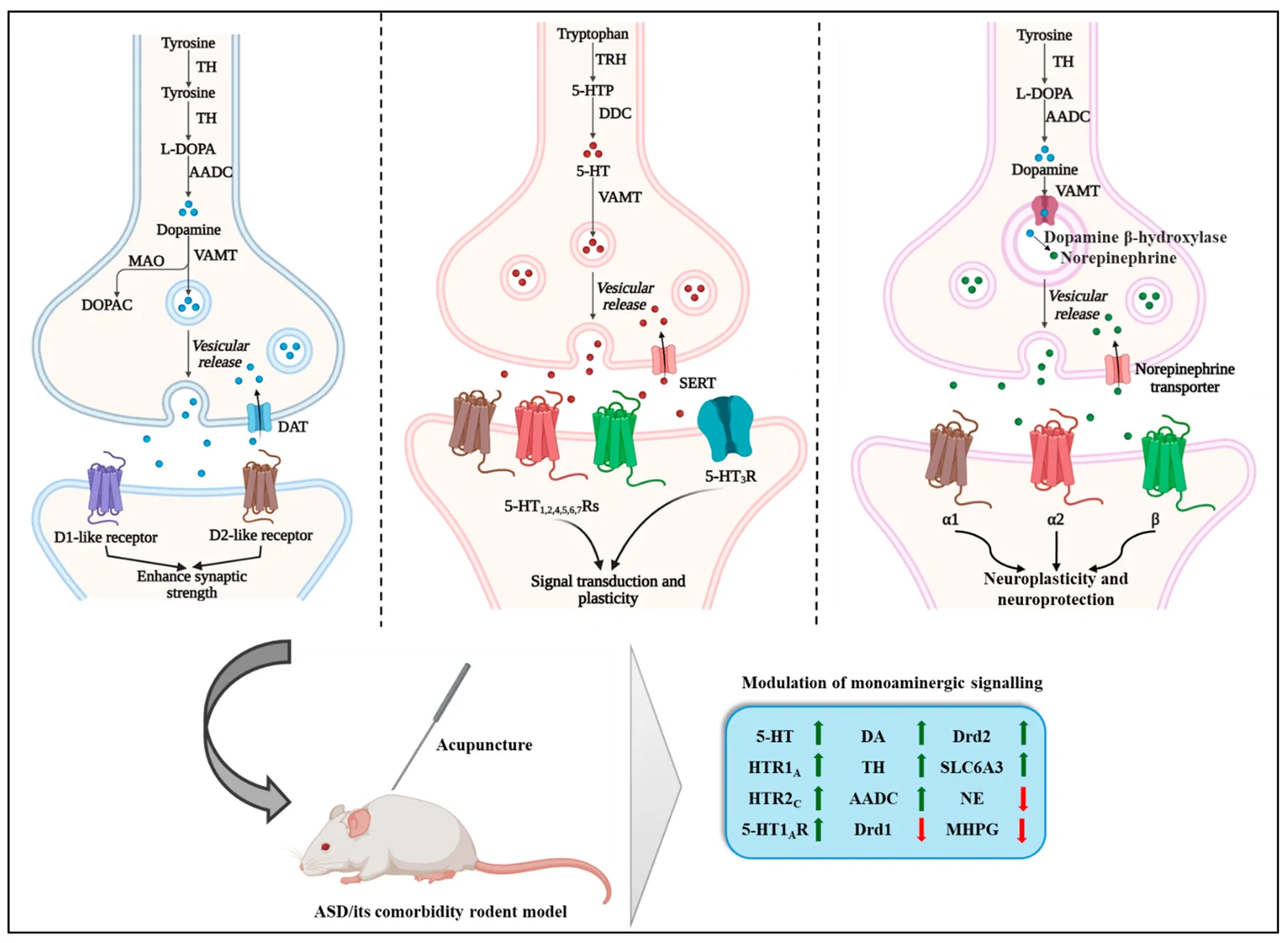
Acupuncture-associated modulation of monoaminergic signaling. 5-HT: 5-Hydroxytryptamine (Serotonin); HTR1A: 5-Hydroxytryptamine Receptor 1A; HTR2C: 5-Hydroxytryptamine Receptor 2C; 5-HT1A(R): 5-Hydroxytryptamine Receptor 1A; DA: Dopamine; TH: Tyrosine Hydroxylase; AADC: Aromatic L-Amino Acid Decarboxylase; Drd1: Dopamine Receptor D1; Drd2: Dopamine Receptor D2; SLC6A3: Solute Carrier Family 6 Member 3; NE: Norepinephrine; MHPG: 3-Methoxy-4-Hydroxyphenylglycol; D1-like receptor: Dopamine D1-like Receptor Family; D2-like receptor: Dopamine D2-like Receptor Family; L-DOPA: L-3,4-Dihydroxyphenylalanine; AADC: Aromatic L-Amino Acid Decarboxylase; MAO: Monoamine Oxidase; VMAT: Vesicular Monoamine Transporter; DOPAC: 3,4-Dihydroxyphenylacetic Acid; TRH: Thyrotropin-Releasing Hormone; DDC: DOPA Decarboxylase. Figure created with BioRender.com.

Additional mechanistic support arises from electroacupuncture stimulation at DU20, HT7, and SP6, which activated cAMP/CREB/BDNF and PI3K/Akt signaling cascades and reduced neuronal apoptosis in the central nervous system [50]. Although investigated primarily in insomnia and depression paradigms, these molecular pathways intersect with monoaminergic regulation, stress responsivity, and synaptic plasticity that also involve noradrenergic circuit alterations described in ASD and related anxiety or mood phenotypes. Preclinical evidence indicates that acupuncture has been associated with modulation of LC–NE-linked signaling dynamics. However, direct demonstration of noradrenergic normalization in ASD clinical populations remains scarce. Key preclinical studies examining monoaminergic and synaptic outcomes after acupuncture in ASD models and related comorbidities are summarized in Table 1 and Figure 3.

## 4. Regulation of Neuroplasticity

Neuroplasticity refers to the nervous system’s remarkable ability to reorganize its structural and functional architecture in response to both internal and external influences. This capacity includes changes in dendritic and synaptic connectivity, long-term potentiation (LTP) and long-term depression (LTD) of synapses, as well as the generation of new neurons (neurogenesis), all of which support learning, memory, and adaptation to experience. Importantly, neuroplasticity is not just a feature of development: even in the adult brain, it is mediated by molecular drivers such as neurotrophic factors, neurotransmitters, growth factors, and inflammatory cytokines, which together orchestrate synaptic remodeling and circuit stabilization.

In ASD and its associated comorbidities, dysregulated neuroplasticity has been increasingly recognized as a central pathological element. Alterations in synaptic scaffolding proteins, imbalance in excitatory/inhibitory signaling, and abnormal expression of neurotrophic molecules have been documented in both preclinical models and clinical populations, indicating impaired synaptic maturation and adaptive rewiring [55]. Evidence further shows that peripheral levels of brain-derived neurotrophic factor (BDNF) and other neurotrophins are often elevated in individuals with ASD compared with neurotypical controls, suggesting compensatory plasticity mechanisms or maladaptive signaling in neurodevelopment [56,57].

Given the critical role of neuroplasticity in behavior, cognition, and network stability, restoring adaptive plasticity represents a promising therapeutic strategy for ASD. Acupuncture has emerged as a candidate intervention, as accumulating data indicate that it can modulate neurotrophins expression, enhance receptor activation, and promote synaptic resilience [58].

### Neurotrophins and Their Receptors

Neurotrophins are a family of growth factors essential for maintaining neuronal survival, synaptic refinement, axonal targeting, and long-term circuit adaptability, which are core processes that collectively define neuroplasticity. Among them, BDNF is the most widely studied due to its high expression in brain regions crucial for learning, memory, and socioemotional regulation, including the hippocampus, PFC, and amygdala [59]. Other neurotrophins, such as neurotrophin-3 (NT-3) and neurotrophin-4/5 (NT-4/5), similarly support synaptic maintenance, dendritic complexity, and activity-dependent remodeling. Neurotrophins actions are primarily mediated through tropomyosin receptor kinase (Trk) receptors, especially TrkB, which is the high-affinity receptor for BDNF [60]. Together, these ligand–receptor systems contribute to structural and functional circuit reorganization underlying learning and adaptive behavior processes that have been reported to be altered in ASD.

**Figure 4:**
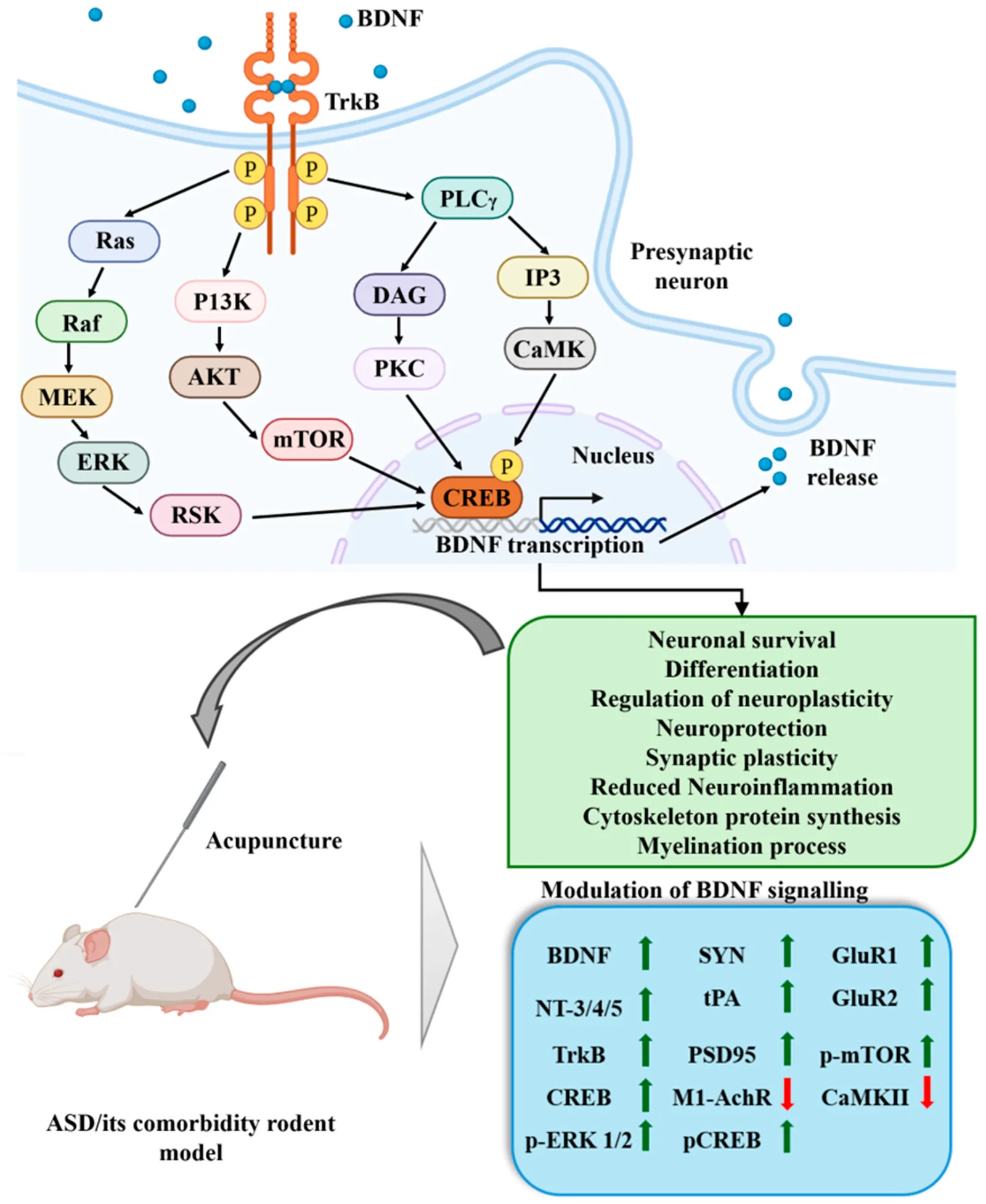
Acupuncture-associated modulation of BDNF signaling. Schematic illustration of brain-derived neurotrophic factor (BDNF) signaling through its high-affinity receptor tropomyosin receptor kinase B (TrkB) and the major intracellular pathways regulating neuronal plasticity. Following synthesis and release, mature BDNF binds to TrkB receptors, inducing receptor phosphorylation and activation of multiple downstream signaling cascades, including the phosphoinositide 3-kinase/protein kinase B (PI3K/AKT) pathway, Ras/Raf/mitogen-activated protein kinase/extracellular signal-regulated kinase (Ras/Raf/MEK/ERK) pathway, and phospholipase Cγ (PLCγ)/diacylglycerol (DAG)/inositol 1,4,5-trisphosphate (IP3) pathway. These signaling cascades activate downstream mediators, including protein kinase C (PKC), calcium/calmodulin-dependent protein kinase (CaMK), mammalian target of rapamycin (mTOR), ribosomal S6 kinase (RSK), and cAMP response element-binding protein (CREB), promoting BDNF gene transcription and sustained neurotrophic signaling. Activation of these pathways contributes to neuronal survival, differentiation, neuroprotection, synaptic plasticity, regulation of neuroplasticity, cytoskeletal protein synthesis, myelination, and reduced neuroinflammation. Experimental studies in autism spectrum disorder (ASD) and related neuropsychiatric models suggest that acupuncture modulates multiple components of the BDNF signaling pathway, including BDNF, TrkB, phosphorylated CREB (pCREB), phosphorylated extracellular signal-regulated kinase 1/2 (pERK1/2), synaptophysin (SYN), tissue plasminogen activator (tPA), postsynaptic density protein 95 (PSD95), metabotropic glutamate receptor 1 (GluR1), metabotropic glutamate receptor 2 (GluR2), phosphorylated mTOR (p-mTOR), M1 muscarinic acetylcholine receptor (M1-AChR), and CaMKII. Ras: Rat Sarcoma Small GTPase; Raf: Rapidly Accelerated Fibrosarcoma Kinase; MEK: Mitogen-Activated Protein Kinase; ERK: Extracellular Signal-Regulated Kinase; NT-3/4/5: Neurotrophin-3/4/5. Figure was created with BioRender.com.

**Figure 5:**
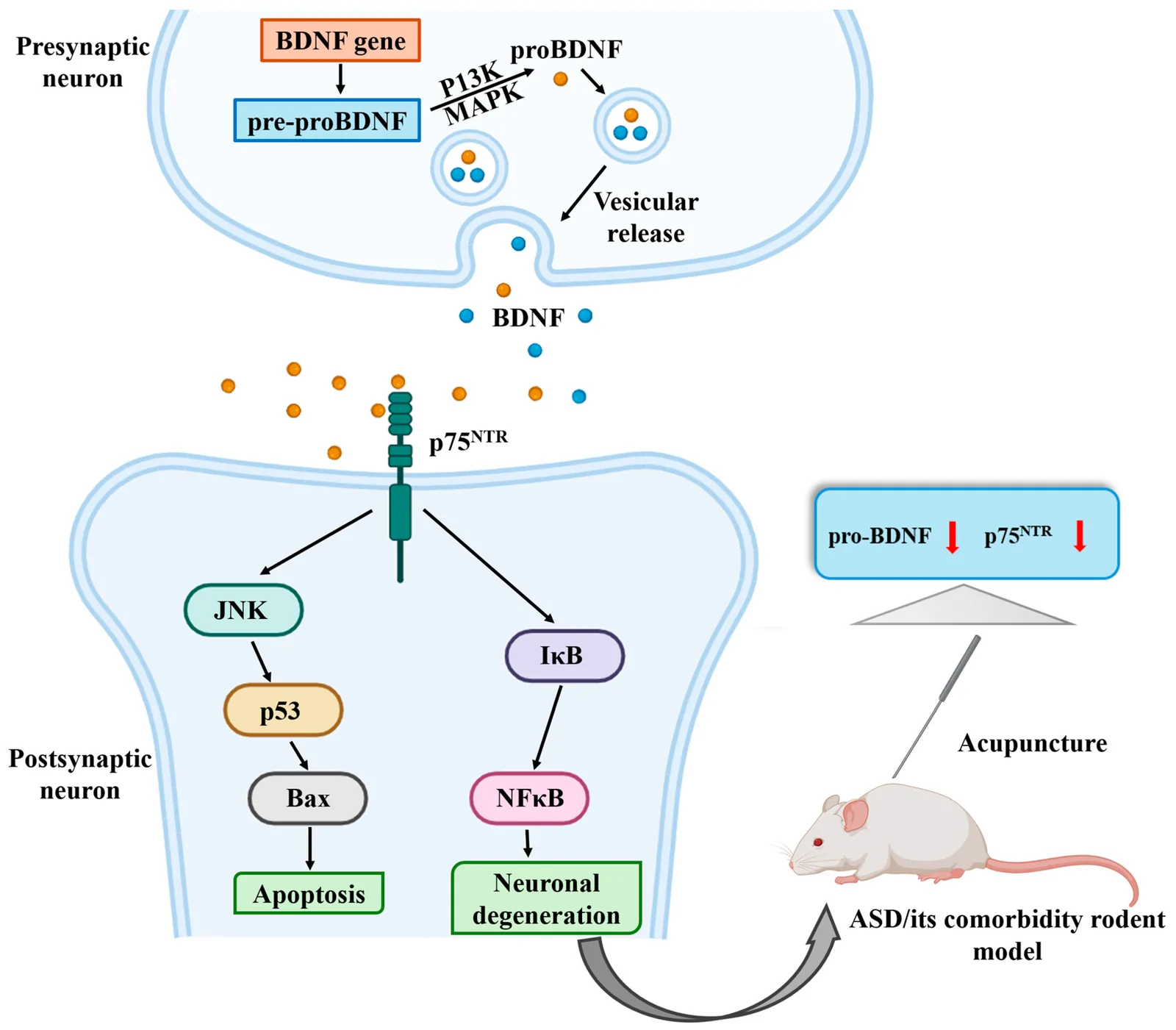
proBDNF–p75NTR signaling pathway and its proposed modulation by acupuncture. Schematic illustration of pro-brain-derived neurotrophic factor (proBDNF) signaling through the p75 neurotrophin receptor (p75NTR). The brain-derived neurotrophic factor (BDNF) gene is transcribed to produce pre-proBDNF, which is subsequently processed into proBDNF before vesicular release. Extracellular proBDNF preferentially binds to p75NTR, activating signaling pathways associated with neuronal apoptosis and neurodegeneration. Binding of proBDNF to p75NTR activates the c-Jun N-terminal kinase (JNK) pathway, leading to sequential activation of p53 and Bcl-2-associated X protein (Bax), thereby promoting apoptotic signaling. In parallel, activation of inhibitor of κB (IκB) and nuclear factor kappa B (NF-κB) signaling contributes to neuronal degeneration and inflammatory responses. Experimental evidence from autism spectrum disorder (ASD) and related neuropsychiatric models suggests that acupuncture may attenuate this pathway by downregulating proBDNF and p75NTR expression, thereby suppressing pro-apoptotic signaling and shifting neurotrophin signaling toward a neuroprotective state that supports neuronal survival and functional recovery. Figure created with BioRender.com.

Under physiological conditions, BDNF binds to TrkB, initiating a cascade of intracellular pathways that collectively regulate plasticity. TrkB activation engages three major downstream axes: (1) the MAPK-ERK pathway, which regulates dendritic growth, long-term potentiation (LTP), and synaptic maturation; (2) the PI3K-Akt signaling pathway, crucial for neuronal survival, spine stabilization, and metabolic homeostasis; and (3) the PLC-γ pathway, which modulates intracellular Ca^2+^ dynamics required for synaptic strengthening. A key convergence point of these pathways is the phosphorylation of cyclic AMP response element-binding protein (CREB), a transcription factor that promotes BDNF gene transcription and drives the synthesis of plasticity-related proteins. Conversely, the stress-activated JNK pathway acts as a negative regulator by promoting neuronal apoptosis, dendritic retraction, and synaptic loss when overactivated [61,62] (Figure 4 and Figure 5). Dynamic balance among these signaling pathways supports plasticity-related processes across cognitive and socioemotional circuits.

Multiple lines of evidence implicate disrupted neurotrophin signaling in ASD [63]. Clinical and preclinical studies report altered peripheral and central BDNF measures, region-specific changes in TrkB expression/activation, and downstream kinase abnormalities (ERK/CREB/PI3K-Akt), although findings are heterogeneous in direction and region because developmental stage, tissue sampled (serum vs brain), and methodology differ across studies (i.e., peripheral BDNF increases in some cohorts versus reductions in others). High-quality reviews on ASD signal transduction emphasize that synaptic scaffold protein anomalies (SHANKs, NRXN/NLGN), excitatory/inhibitory imbalance, and impaired activity-dependent trophic signaling form an interconnected pathological triad in ASD models and patient tissue. In short: the neurotrophin–TrkB axis is plausibly implicated in ASD pathophysiology, but the literature is noisy and not yet definitive about consistent directional changes across all brain regions or clinical subtypes [64].

This unevenness carries over into how acupuncture appears to act on BDNF. Rather than producing a blanket increase wherever it is measured, the effect looks state- and region-dependent: in stress or depletion models, where baseline BDNF is already low, acupuncture tends to raise it, while effects in already-inflamed or hyperactive states are less consistent. Even within a single study, results can diverge by brain region. Electroacupuncture raised tPA, BDNF, and TrkB expression in the hippocampus of chronically stressed rats but left proBDNF and p75NTR unchanged, and BDNF in the raphe nuclei of the same animals fell rather than rose [65]. If acupuncture’s effect on BDNF in ASD follows a similar pattern, its net impact would depend on an individual’s baseline neurotrophin status, developmental stage, and the specific brain region examined, rather than acting as a uniform upregulator.

A further layer of complexity, largely unaddressed in the acupuncture–ASD literature so far, is epigenetic regulation of the BDNF gene itself. BDNF transcription is sensitive to activity-dependent DNA methylation and histone modification at its promoter, and these marks can persist well beyond the initial stimulus, shaping plasticity capacity over the longer term. Whether acupuncture engages these mechanisms at the BDNF locus, in ASD or in the comorbid conditions reviewed here, has not to our knowledge been tested directly, and represents a natural next step for mechanistic work in this area.

Experimental evidence from the last decade indicates that acupuncture recruits neurotrophin-based mechanisms that are directly relevant to plasticity restoration. In children with ASD, laser acupuncture applied to neurodevelopment-related scalp and body acupoints produced biochemical improvements accompanied by modulation of circulating neurotrophic factors, suggesting restoration of plasticity-associated molecular balance [58]. In a valproic acid (VPA)-induced rat model of ASD, transcutaneous electrical acupoint stimulation (TEAS) altered the amygdala transcriptome, with RNA sequencing revealing enrichment of genes involved in neuronal development and the neurotrophin signaling pathway. These findings suggest that TEAS may promote neuroplasticity and support neuronal survival through modulation of neurotrophic signaling in ASD [66]. In chronic stress-induced depression models, electroacupuncture enhanced hippocampal BDNF levels through activation of the tPA/plasmin cleavage pathway, facilitating conversion of pro-BDNF to mature BDNF and improving synaptic resilience and behavioral outcomes [65]. MA at HT7 restored stress-induced behavioral abnormalities while normalizing limbic–prefrontal neurotransmission and enhancing neurotrophic support, highlighting coordinated BDNF-dependent circuit remodeling [18]. Additional experimental evidence showed that acupuncture increased hippocampal BDNF expression through modulation of corticosterone and sex hormone levels, linking endocrine regulation to synaptic plasticity mechanisms in stress-related comorbidities [67]. Further clinical/preclinical evidence supporting acupuncture-mediated modulation of neurotrophins and related pathways in ASD and related comorbidities is systematically summarized in Table 2. Collectively, these findings indicate that acupuncture promotes adaptive neuroplastic remodeling by regulating BDNF synthesis, maturation, and epigenetic control across ASD-relevant pathological conditions.

## 5. Discussion

Drawing exclusively from studies published between 2015 and 2025, this review provides an updated synthesis of acupuncture-associated neuro-modulatory mechanisms within an ASD-relevant systems framework. This review integrates evidence across neurotransmitter regulation, excitatory–inhibitory balance, monoaminergic signaling, and neurotrophin-mediated plasticity within a unified neurobiological framework relevant to ASD. Rather than evaluating acupuncture as a disorder-specific intervention, we evaluate whether acupuncture-associated molecular and circuit changes converge with neurobiological processes implicated in ASD. This integrative perspective shifts the discussion from symptom-based extrapolation toward pathway-level analysis of transdiagnostic neural mechanisms.

### 5.1. Preclinical Models

ASD is associated with widespread neurobiological alterations that extend across multiple signaling systems rather than arising from a single molecular abnormality. Converging evidence indicates that disturbances in neurotransmitter regulation, imbalance between excitatory and inhibitory transmission, altered monoaminergic activity, and deficits in adaptive neuroplastic processes collectively contribute to atypical brain development. Disrupted plasticity is increasingly viewed as a central feature of ASD, influencing synaptic formation, neuronal connectivity, and the maturation of cortical–limbic circuits that support social behavior and cognitive function. Abnormal interactions among glutamatergic, GABAergic, and monoaminergic networks can impair synaptic strengthening and experience-dependent circuit refinement, leading to reduced behavioral flexibility and challenges in social and adaptive domains. Within this framework, the emerging evidence reviewed here suggests that acupuncture may exert therapeutic effects in ASD by restoring neurotrophin-dependent neuroplastic mechanisms.

Although ASD is a heterogeneous neurodevelopmental condition for which a complete cure remains unlikely in the near future, therapeutic strategies that enhance adaptive functioning and improve quality of life hold substantial clinical and societal value. Evidence suggesting that acupuncture can modulate neural plasticity, regulate emotional processing, and reduce comorbid symptoms such as anxiety, depression, and sleep disturbances indicates its potential as a complementary intervention aimed at functional improvement rather than disease eradication. From a translational perspective, even modest improvements in social engagement, emotional regulation, sensory processing, or daily living skills could significantly reduce caregiver burden and enhance long-term well-being for individuals with ASD and their families.

One of the most consistent findings across preclinical studies is the capacity of acupuncture to modulate neurotransmitter dynamics. Acupuncture has been shown to influence glutamate and GABA signaling, which are central to maintaining E/I balance within cortical and limbic circuits implicated in ASD. Restoration of inhibitory tone through regulation of GABAergic enzymes and transporters, alongside normalization of glutamatergic transmission, may reduce neuronal hyperexcitability and improve network synchronization. These changes are particularly relevant given the well-documented shift toward excitation in ASD models, which contributes to sensory hypersensitivity, repetitive behaviors, and impaired cognitive flexibility.

Beyond classical excitatory and inhibitory neurotransmission, acupuncture also modulates monoaminergic pathways, including serotonergic, dopaminergic, and noradrenergic systems. These pathways play essential roles in mood regulation, reward processing, executive function, and social behaviors—domains frequently disrupted in ASD and its comorbidities such as anxiety and depression. Evidence from both animal models and human studies demonstrates that acupuncture can normalize serotonin turnover, regulate dopamine synthesis and receptor expression, and influence stress-related neuroendocrine responses. Through these mechanisms, acupuncture may recalibrate corticolimbic circuits involved in emotional regulation and motivation, thereby contributing to improvements in behavioral outcomes.

Neurotrophins represent an additional mechanistic layer linking neurotransmitter modulation to long-term synaptic remodeling. Altered levels of BDNF and related growth factors have been implicated in abnormal synaptic pruning and dendritic development in ASD. Studies reviewed here indicate that acupuncture can influence neurotrophin expression and signaling pathways associated with neuronal survival, synaptogenesis, and activity-dependent plasticity. Importantly, neurotrophin regulation appears to interact with monoaminergic and glutamatergic systems, suggesting that acupuncture-induced increases in BDNF may stabilize synaptic changes initiated by neurotransmitter modulation. In this context, neurotrophins may function as downstream mediators that consolidate circuit-level adaptations rather than as isolated therapeutic targets.

An integrative interpretation of these findings supports the concept that acupuncture acts as a network-level neuro-modulatory intervention. Rather than targeting a single molecular pathway, acupuncture appears to orchestrate coordinated adjustments across neurotransmitter systems, neuroendocrine responses, and neuroplastic signaling networks. This systems-level regulation may explain why acupuncture demonstrates potential benefits across diverse ASD-related domains, including social interaction, emotional regulation, sleep disturbances, and cognitive performance. The convergence of effects on E/I balance, monoaminergic signaling, and neurotrophins pathways underscores the interconnected nature of ASD neurobiology and highlights acupuncture’s potential to restore functional equilibrium within distributed neural circuits.

### 5.2. Human Trials

Clinical evidence supporting the neuro-modulatory mechanisms described above remains considerably more limited than the preclinical literature. Clinical studies investigating acupuncture in children with ASD have reported improvements in behavioral outcomes, including reductions in autism severity and improvements in communication, social interaction, and adaptive functioning, as assessed using standardized instruments such as the Childhood Autism Rating Scale (CARS), Autism Behaviour Checklist (ABC), and Autism Treatment Evaluation Checklist (ATEC) [72,73,74]. In addition, pilot and feasibility studies have demonstrated that acupuncture and related interventions are generally well tolerated and feasible in pediatric ASD populations [75]. However, only a limited number of clinical studies have incorporated objective molecular or neurochemical outcome measures alongside behavioral assessments.

For example, it is demonstrated that low-level laser acupuncture modulated circulating biomarkers by reducing plasma BDNF levels and altering miR-320 expression in children with ASD [58], suggesting that acupuncture may influence neuroplasticity-related molecular pathways. Nevertheless, these clinical studies remain scarce, and the biomarkers investigated represent only a small subset of the neurotransmitter, neurotrophin, and neuroimmune pathways identified in preclinical studies. Consequently, the mechanistic framework presented in this review is supported predominantly by preclinical evidence and requires further validation through well-designed clinical studies integrating behavioral, molecular, and neurophysiological outcome measures.

Despite promising evidence, significant limitations remain. A large proportion of mechanistic data derives from rodent models, and translational validation in human populations remains limited. Additionally, variability in acupuncture protocols—including differences in acupoints, stimulation parameters, and treatment duration—poses challenges for reproducibility and mechanistic interpretation. Many clinical studies also lack standardized neurochemical or electrophysiological outcome measures, making it difficult to directly link molecular changes to behavioral improvements. Furthermore, ASD heterogeneity complicates interpretation, as neurotransmitter and neurotrophin alterations may differ across developmental stages and clinical subtypes.

### 5.3. Future Directions

Future research should adopt integrative, multimodal designs that simultaneously assess neurotransmitter dynamics, E/I balance, monoaminergic function, and neurotrophin signaling in response to acupuncture. Combining molecular analyses with functional neuroimaging, electrophysiological recordings, and behavioral assessments will be critical for establishing causal relationships between neuro-modulatory changes and clinical outcomes. Standardization of acupuncture protocols and incorporation of well-defined mechanistic biomarkers will also enhance reproducibility and facilitate meta-analytic comparisons. Additionally, exploring interactions between neural, immune, and metabolic pathways may provide deeper insight into how acupuncture influences systemic contributors to ASD pathophysiology.

Another important consideration is the inherently holistic nature of acupuncture treatment. Traditional and modern clinical practice rarely rely on stimulation of a single acupoint; instead, therapeutic effects are thought to arise from coordinated activation of multiple acupoints that collectively influence distributed neural networks. However, many experimental studies employ simplified single-point protocols to facilitate mechanistic interpretation, which may not fully capture the synergistic neurobiological effects observed in clinical settings. Given that ASD involves widespread network-level dysfunction, future research should investigate multi-acupoint strategies and their potential synergistic effects on large-scale neural circuits, neurotransmitter systems, and neuroplastic signaling pathways. Comparative studies evaluating single versus combined acupoint protocols, integrated with neuroimaging, electrophysiology, and molecular analyses, could provide critical insight into how acupuncture modulates complex brain networks relevant to ASD symptomatology.

Clinical translation also requires attention to practical delivery. Repeated needle insertion may be poorly tolerated in children with ASD-associated sensory hypersensitivity, and feasibility data on repeated-session protocols in this population remain limited [75]. The choice between manual and electroacupuncture, and the corresponding stimulation parameters, has largely been determined empirically rather than through direct comparative trials, and standardized, validated sham-control procedures are not yet established for pediatric ASD studies. These practical and methodological gaps will need to be addressed before the mechanistic findings summarized here can inform clinical protocols.

Given the substantial clinical and biological heterogeneity of ASD, future acupuncture research should also incorporate stratified study designs that account for ASD subtype, comorbidity profile, and biomarker status (e.g., baseline GABA/glutamate ratio, BDNF level, or inflammatory markers). Such stratification would clarify whether acupuncture-responsive neurobiological subgroups exist within the broader ASD population and would help resolve the heterogeneous BDNF and monoaminergic findings summarized in this review.

### 5.4. Limitations

Several limitations should be considered when interpreting the findings presented in this review. First, this work represents a qualitative narrative synthesis rather than a systematic review or meta-analysis; therefore, the conclusions are based on descriptive integration of available studies rather than quantitative effect estimation. Consistent with the narrative approach, a formal risk-of-bias assessment was not performed, and studies reporting null or opposing findings may be under-represented in the preclinical acupuncture literature; this potential selection bias should be considered when interpreting the consistency of effects summarized here. Second, a substantial proportion of mechanistic evidence originates from animal models, particularly rodent studies employing valproic acid-induced ASD, stress, depression, epilepsy, or insomnia paradigms. While these models provide valuable insight into molecular and circuit-level mechanisms, their direct translation to human ASD populations remains uncertain. Third, considerable heterogeneity exists across included studies in terms of acupuncture protocols, including acupoint combinations, stimulation parameters, treatment duration, and outcome measures, which limits direct comparison and may introduce interpretative bias. Fourth, relatively few studies have directly investigated acupuncture mechanisms specifically within ASD populations; much of the mechanistic framework presented here is inferred from related neuropsychiatric or neurodevelopmental conditions that share overlapping pathophysiological pathways.

## 6. Conclusions

This review provides an integrative mechanistic overview of how acupuncture may influence core neurobiological processes relevant to autism spectrum disorder and its associated comorbidities. Converging preclinical and clinical evidence indicates that acupuncture modulates multiple neurobiological pathways, encompassing E/I balance, monoaminergic transmission, and neurotrophin-dependent plasticity, with implications for ASD-relevant domains including social cognition, emotional regulation, and sensory processing. However, current evidence remains preliminary and is largely derived from preclinical models and small-scale clinical studies. Rigorous translational research incorporating standardized acupuncture protocols, multimodal biomarker assessment, and well-designed clinical trials will be essential to determine therapeutic efficacy and clarify the precise neural mechanisms involved. As understanding of ASD neurobiology continues to evolve, acupuncture may emerge as a candidate intervention with defined molecular targets aimed at enhancing neural adaptability and improving functional outcomes rather than providing disease-modifying treatment.

## Figures and Tables

**Table 1:** Acupuncture effects on monoaminergic/related markers in ASD and its comorbidities (selected representative studies from 2015–2025).

Reference	Model/Participants	Relevance to ASD	Acupoints/Stimulation	Type/Regimen	Brain Region/Sample Studied	Session/Duration	Main Markers Measured	Main Molecular Findings
[15]	VPA-induced rat model of ASD	ASD model	Shéntíng (GV24) and bilateral Běnshén (GB13)	MA	Hippocampus	40 min with manual twisting of the needles every 10 min, 5 days per week, with 2 days off for 4 weeks	5-HT, Tph1	upregulated serotonergic genes (Tph1) and increased hippocampal serotonin levels
[48]	Acute restraint stress model of SD male rat	Comorbidity model (acute restraint stress)	PC6 (Neiguan), HT7 (Shenmen)	MA	central nucleus of the amygdala	1 min/session, once daily for 3 days (total 3 treatments)	NE and MHPG	Reduced elevated NE and its metabolite MHPG
[49]	CUMS model of C57BL/6 male mice	Comorbidity model (Depression)	KI10·LR8·LU8·LR4	EA	hippocampus	needles inserted 3–4 mm, rotated (~2 rotations/s) for 30 s and removed immediately; administered daily for 2 weeks	5-HT and NE	elevated secretion of 5-HT and NE
[50]	Chlorophenylalanine-induced insomnia model of SD male rat	Comorbidity model (insomnia)	Baihui bilateral Shen Men, and bilateral Sanyinjiao	EA	hippocampus and brainstem	30 min/session, once daily for 5 days (2/15 Hz, 1 mA sparse–dense waveform)	5-HT, DA, Epinephrine (EPI), and NE	higher levels of 5-HT and lower levels of DA, NE, and EPI
[51]	CUMS model of Sprague–Dawley (SD) male rat	Comorbidity model (Depression)	Shangxing (GV23) and Fengfu (GV16)	MA	Serum	Needles inserted to ~5 mm at oblique angles (GV16: ~30°, GV23: ~15°) and retained for 20 min; treatments administered every other day for 4 weeks	5-HT	elevated secretion of 5-HT
[52]	CUMS model of SD male rat	Comorbidity model (Depression)	GV23 and Daling		Hippocampus and serum	Daily acupuncture administered 1 h before CUMS during week 4 at Shangxing and Daling (≈2–5 mm insertion; oblique at Shangxing, vertical at Daling); needles retained for 20 min with manual rotation every 5 min	DA and 5-HT	elevated secretion of DA and 5-HT
[53]	CUMS model of Wistar rats	Comorbidity model (chronic unpredictable mild stress)	GV 20 and GV 29	EA	Hippocampus	once daily for 30 min using 2 Hz, 0.6 mA continuous stimulation for 14 days	5-HT1A and 5-HT1B receptors	Improvement in mRNA and protein expression of 5-HT1A and 5-HT1B receptor and the morphologies of hippocampal organelles and synapses
[54]	CUMS model of SD male rat	Comorbidity Model (Depression)	*Yintang* (EX-HN3) and *Baihui* (DU20)	EA	hippocampus	sparse waves at 2 Hz and 0.6 mA for 30 min, once daily for 14 days	5-HT, NE, Glu, and GABA	significantly higher levels of hippocampal 5-HT, upregulated Glu, and GABA mRNA expressions

**Table 2:** Acupuncture effects on neurotrophin signaling/related markers in ASD and its comorbidities (selected 2015–2025 studies).

Reference	Model/Participants	Relevance to ASD	Acupoints/Stimulation	Type/Regimen	Brain Region/Sample Studied	Session/Duration	Main Markers Measured	Main Molecular Findings
[50]	Chlorophenylalanine-induced insomnia model of SD male rat	Comorbidity model (insomnia)	Baihui bilateral Shen Men, and bilateral Sanyinjiao	EA	Hippocampus and brainstem	30 min/session, once daily for 5 days (2/15 Hz, 1 mA sparse–dense waveform)	TrkB, PI3K, Akt, P-TrkB, p-Akt, cAMP, CREB, BDNF, and brain apoptosis markers	Upregulation in the mRNA and protein expression levels of p-TrkB, PI3K p-Akt, cAMP, CREB, BDNF, and down regulated brain apoptosis markers
[51]	CUMS model of SD male rat	Comorbidity Model (Depression)	Shangxing (GV23) and Fengfu (GV16)	MA	Lateral Habenular tissue	Needles inserted to ~5 mm at oblique angles (GV16: ~30°, GV23: ~15°) and retained for 20 min; treatments administered every other day for 4 weeks	pro-BDNF, SYN, BDNF, TrkB and CREB	Elevated expression of pro-BDNF and SYN proteins, BDNF, TrkB and CREB mRNA and protein
[54]	CUMS model of SD male rat	Comorbidity Model (Depression)	Yintang (EX-HN3) and Baihui (DU20)	EA	hippocampus	sparse waves at 2 Hz and 0.6 mA for 30 min, once daily for 14 days	TrkB, BDNF, pCREB, CREB, PKA, CaMKI, and Akt	Elevation in TrkB, pCREB, and BDNF protein levels and decrease in CaMKII levels in hippocampus
[58]	Children with ASD	ASD patients	Multiple scalp and body acupoints, including GV20, EX-HN1, GV24, GB20, GV26, EX-HN3 (four surrounding points around GV20), bilateral LI4, ST36, SP6, and the scalp speech region	Low-level laser acupuncture	Plasma	LLLA therapy twice a week for 12 sessions	BDNF levels, and miR-320	Reduction in plasma BDNF level
[65]	CUMS model of SD male rat	Comorbidity Model (Depression)	Baihui (DU20) and Yintang (EX-HN3)	EA	Hippocampus and raphe nuclei	20 min of EA (2 Hz, 2 mA) daily	BDNF, TrkB, tissue plasminogen activator (tPA), proBDNF, and p75NTR	increased the expression of tPA, BDNF, TrkB, and BDNF mRNA and concentrations of BDNF and TrkB protein and decreased proBDNF, and p75^NTR^ protein concentrations in the hippocampus increased concentration of tPA in raphe nuclei
[66]	VPA-induced Wistar rat model of ASD	ASD model	Bilateral ST36 (Zusanli)	Transcutaneous electrical acupoint stimulation	Hypothalamus	30 min/session, once daily for 7 days (PND7–PND13; 2/15 Hz, 2–4 mA sparse–dense waveform)	RNA-seq, GO and KEGG pathway analyses	Upregulated neurotrophic signalling pathway and genes involved in neuronal development, suggesting improved neurodevelopment in VPA-induced ASD rats.
[67]	Post-weaning social isolation model of SD male rats	Comorbidity Model (Depression)	Baihui (DU 20), Yintang (EX-HN3), Shenshu (BL 23), Pishu (BL 20), Ganshu (BL 18), Xinshu (BL 15) and Guanyuan (Ren 4)	MA	Hippocampus	3 weeks	BDNF	Elevation in hippocampal BDNF protein expression
[68]	Forced swimming-induced depression mouse model	Comorbidity Model (Depression)	GV20 and *Yintang*	MA	right anterior cerebrum		NGF, BDNF, NT-3, and NT-4/5	acupuncture at GV20 + *Yintang* elevated the expression of BDNF, NT-3, and NT-4/5
[69]	Forced swimming stress test (FST) model mice and CUMS model of SD male rat	Comorbidity Model (Depression)	GU20, GV29, LI4, LR3	MA	prefrontal cortex	Single acupuncture session (5 mm insertion depth) administered for 20 min on day 3 following drug treatment	M1-AchR, AMPA receptors (GluR1 and GluR2), BDNF, mTOR, p-mTOR, synapsin I, and PSD95	inhibited the expression of M1-AchR and promoted the expression of GluR1, GluR2, BDNF, p-mTOR, synapsin I, PSD95 in both the models
[70]	CUMS model of SD male rat	Comorbidity Model (Depression)	GV 20 and GV 29	MA	Serum and hippocampus	20 min per session, once daily, for 28 days	BDNF, acH3K9 and HDAC2	Restored reduced BDNF levels in both serum and hippocampal tissue, along with normalization of BDNF mRNA expression
[71]	CUMS model of SD male rat	Comorbidity Model (Depression)	GV 20 and GV 29	MA	prefrontal cortex	for 10 min, once daily for 21 days	p-ERK 1/2 and BDNF	Upregulation of p-ERK 1/2 and BDNF expression

## Data Availability

All data generated or analyzed during this study are included in this published article and its referenced literature sources.

## Supplementary Materials

The following supporting information can be downloaded at: https://www.mdpi.com/article/10.3390/biomedicines14081701/s1, Figure S1: Acupuncture-associated modulation of excitatory–inhibitory signalling pathways.

## Abbreviations

The following abbreviations are used in this manuscript:

ASD	Autism Spectrum Disorder
ADHD	Attention Deficit/Hyperactivity Disorder
GLU	Glutamate
GABA	γ-aminobutyric acid
MRS	Magnetic Resonance Spectroscopy
PV	Parvalbumin
mPFC	Medial Prefrontal Cortex
CUMS	Chronic Unpredictable Mild Stress
EA	Electroacupuncture
MA	Manual Acupuncture
GAD65	Glutamate Decarboxylase 65
GAD67	Glutamate Decarboxylase 67
GABA-T	γ-Aminobutyric Acid Transaminase
EAATs	Excitatory Amino Acid Transporters
NMDAR	N-Methyl-D-Aspartate Receptor
GABAAR	γ-Aminobutyric Acid Type A Receptor
GABABR	γ-Aminobutyric Acid Type B Receptor
ErbB4	Erb-B2 Receptor Tyrosine Kinase 4
NRG1	Neuregulin 1
MGluR	Metabotropic Glutamate Receptor
AMPAR	α-Amino-3-hydroxy-5-methyl-4-isoxazolepropionic Acid Receptor
KAR	Kainate Receptor
GAT-1	γ-Aminobutyric Acid Transporter 1
SV2A	Synaptic Vesicle Glycoprotein 2A
NE	Norepinephrine
LC	Locus Coeruleus
VPA	Valproic Acid
SD	Sprague–Dawley
5-HT	5-Hydroxytryptamine (Serotonin)
HTR1A	5-Hydroxytryptamine Receptor 1A
HTR2C	5-Hydroxytryptamine Receptor 2C
5-HT1A(R)	5-Hydroxytryptamine Receptor 1A
DA	Dopamine
TH	Tyrosine Hydroxylase
Drd1	Dopamine Receptor D1
Drd2	Dopamine Receptor D2
SLC6A3	Solute Carrier Family 6 Member 3
MHPG	3-Methoxy-4-Hydroxyphenylglycol
D1-like receptor	Dopamine D1-like Receptor Family
D2-like receptor	Dopamine D2-like Receptor Family
L-DOPA	L-3,4-Dihydroxyphenylalanine
AADC	Aromatic L-Amino Acid Decarboxylase
MAO	Monoamine Oxidase
VMAT	Vesicular Monoamine Transporter
DOPAC	3,4-Dihydroxyphenylacetic Acid
TRH	Thyrotropin-Releasing Hormone
DDC	DOPA Decarboxylase
LTP	Long-Term Potentiation
LTD	Long-Term Depression
NT-3/4/5	Neurotrophin-3/4/5
BDNF	Brain-Derived Neurotrophic Factor
pro-BDNF	Precursor Brain-Derived Neurotrophic Factor
TrkB	Tropomyosin Receptor Kinase B
CREB	cAMP Response Element-Binding Protein
p-CREB	Phosphorylated cAMP Response Element-Binding Protein
p-ERK1/2	Phosphorylated Extracellular Signal-Regulated Kinase 1 and 2
SYN	Synapsin
tPA	Tissue Plasminogen Activator
PSD95	Postsynaptic Density Protein 95
M1-AChR	Muscarinic Acetylcholine Receptor M1
GluR1	Glutamate Receptor 1
GluR2	Glutamate Receptor 2
p-mTOR	Phosphorylated Mechanistic Target of Rapamycin
CaMKII	Calcium/Calmodulin-Dependent Protein Kinase II
p75NTR	p75 Neurotrophin Receptor
JNK	c-Jun N-terminal Kinase
p53	Tumor Protein p53
Bax	Bcl-2-Associated X Protein
IκB	Inhibitor of Nuclear Factor Kappa B
NF-κB	Nuclear Factor Kappa B
Ras	Rat Sarcoma Small GTPase
Raf	Rapidly Accelerated Fibrosarcoma Kinase
MEK	Mitogen-Activated Protein Kinase
ERK	Extracellular Signal-Regulated Kinase
RSK	Ribosomal S6 Kinase
PI3K	Phosphoinositide 3-Kinase
Akt	Protein Kinase B
mTOR	Mechanistic Target of Rapamycin
PLC-γ	Phospholipase C Gamma
DAG	Diacylglycerol
PKC	Protein Kinase C
IP3	Inositol 1,4,5-Trisphosphate
CaMK	Calcium/Calmodulin-Dependent Protein Kinase
MAPK	Mitogen-Activated Protein Kinase
