# Supplementary material for: Acupuncture in Autism Spectrum Disorder: A Narrative Review of Neurotransmitter Regulation and Neuroplasticity
Source: Biomedicines. 2026 Jul 29;14(8):1701. doi: 10.3390/biomedicines14081701 (PMC13510257; doi:10.3390/biomedicines14081701)

Figure S1. Acupuncture-associated modulation of excitatory–inhibitory signalling pathways. GAD65, Glutamate Decarboxylase 65; GAD67, Glutamate Decarboxylase 67; GABA-T, Gamma-Aminobutyric Acid Transaminase; GABA, Gamma-Aminobutyric Acid; EAATs, Excitatory Amino Acid Transporters; NMDAR, N-Methyl-D-Aspartate Receptor; Glu, Glutamate; GABA$_A$R, Gamma-Aminobutyric Acid Type A Receptor; GABA$_B$R, Gamma-Aminobutyric Acid Type B Receptor; ErbB4, Erb-B2 Receptor Tyrosine Kinase 4; NRG1, Neuregulin 1; mGluR, Metabotropic Glutamate Receptor; AMPAR, α-Amino-3-hydroxy-5-methyl-4-isoxazolepropionic Acid Receptor; KAR, Kainate Receptor; GAT-1, Gamma-Aminobutyric Acid Transporter 1; and SV2A, Synaptic Vesicle Glycoprotein 2A. Figure created with BioRender.com.

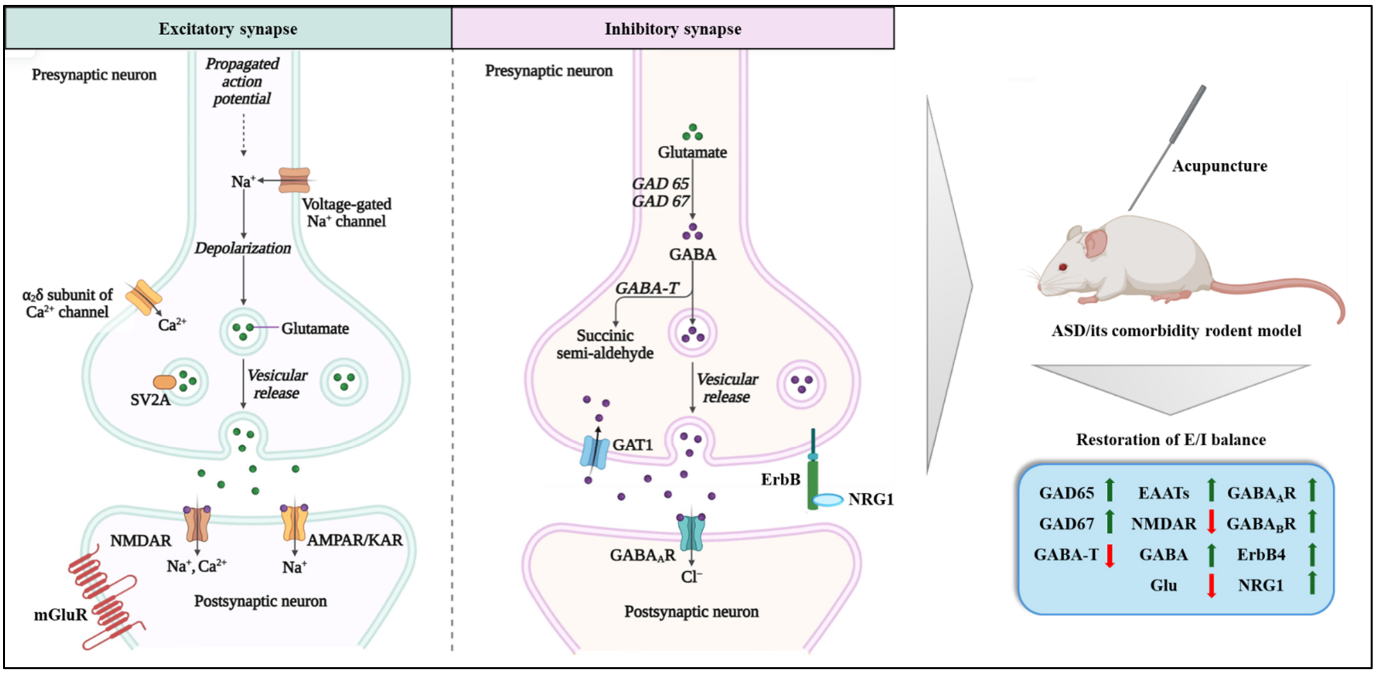

Supplement: Supplementary file 1 [file biomedicines-14-01701-s001.zip › biomedicines-4421592-supplementary.pdf]
